# Can Neural Activation in Dorsolateral Prefrontal Cortex Predict Responsiveness to Information? An Application to Egg Production Systems and Campaign Advertising

**DOI:** 10.1371/journal.pone.0125243

**Published:** 2015-05-27

**Authors:** Brandon R. McFadden, Jayson L. Lusk, John M. Crespi, J. Bradley C. Cherry, Laura E. Martin, Robin L. Aupperle, Amanda S. Bruce

**Affiliations:** 1 Department of Food and Resource Economics, University of Florida, Gainesville, Florida, United States of America; 2 Department of Agricultural Economics, Oklahoma State University, Stillwater, Oklahoma, United States of America; 3 Department of Agricultural Economics, Kansas State University, Manhattan, Kansas, United States of America; 4 Department of Behavioral Pediatrics at the University of Kansas Medical Center, Kansas City, Kansas, United States of America; 5 Department of Preventive Medicine and Public Health, University of Kansas Medical Center, Kansas City, Kansas, United States of America; 6 Laureate Institute for Brain Research, University of Tulsa, Tulsa, Oklahoma, United States of America; Radboud University Nijmegen, NETHERLANDS

## Abstract

Consumers prefer to pay low prices and increase animal welfare; however consumers are typically forced to make tradeoffs between price and animal welfare. Campaign advertising (i.e., advertising used during the 2008 vote on Proposition 2 in California) may affect how consumers make tradeoffs between price and animal welfare. Neuroimaging data was used to determine the effects of brain activation in dorsolateral prefrontal cortex (dlPFC) on choices making a tradeoff between price and animal welfare and responsiveness to campaign advertising. Results indicated that activation in the dlPFC was greater when making choices that forced a tradeoff between price and animal welfare, compared to choices that varied only by price or animal welfare. Furthermore, greater activation differences in right dlPFC between choices that forced a tradeoff and choices that did not, indicated greater responsiveness to campaign advertising.

## Introduction

The goal of neuroeconomics is to supplement traditional economic models by providing a mechanistic explanation of how choices are made [1, 2]. A better understanding of why choices are made may improve predictions of choices and responses to information. In the case of multi-attribute choice, economists have proposed several models, such as the random utility and expected utility models, to describe how consumers arrive at a given choice (the interested reader is referred to the discussion and literature in [3]). Findings from neuroscience have given empirical support for these theoretical constructs. Specifically, studies have shown that individuals assign values to individual attributes and sum the values for each option [4–7]. Values of each option are compared and an optimal choice is made by choosing the option that provides the greatest value.

The dorsolateral prefrontal cortex (dlPFC) is an area of the brain involved in cognitive control. Previous research demonstrates that dlPFC is engaged in reward processing [8] and plays a causal role in the computation of values during decision-making [5, 9]. Wallis and Miller [10] concluded that dlPFC likely encoded to-be-delivered reward values and made comparisons between two different reward amounts. Thus, it is likely that dlPFC plays an important role when making a decision between choices that vary by multiple attributes.

Additionally, previous research has examined the role of dlPFC in food choices and dlPFC has also been identified as a correlate with willingness-to-pay (WTP) for food [11, 12]. Linder et al. [7] evaluated neural activity for food labeled organic versus conventionally produced and determined that dlPFC showed increased activity during the presentation of the organic label. However, participants in Linder et al. [7] did not make choices between two options that varied by production label and price. Thus, a decision was not made, and more importantly, a tradeoff between attributes was not forced.

A forced tradeoff between attributes is important because neuroeconomic research has shown that multi-attribute choices with conflicting individual attribute values increase the uncertainty of value prediction [6]. For example, people typically prefer food production methods that are viewed as more animal friendly *and* lower prices; however, this is not a realistic option. In the marketplace, people are often forced to make tradeoffs between individual attributes. The conflict between individual attributes increases uncertainty of value prediction for a choice decision when there is not an overwhelming preference for one attribute over another. Previous research has concluded dlPFC may encode uncertainty in valuation of choices [6]. Furthermore, a recent study demonstrated that dlPFC showed greater activation when passively viewing multiple food product attributes [13]. Given the role of dlPFC in reward anticipation and value computation, dlPFC may also play a role in multi-attribute decision making, and in resolving situations in which an individual must make a tradeoff between attributes.

If a person has a strong preference for lower prices or cage free eggs, then it is unlikely that information (i.e., campaign advertising) will have much influence on their decision. By contrast, a consumer who is closer to indifference, who is more unsure about whether they are willing to pay a premium for cage free eggs, is likely to be more influenced by information. Positive or negative information might tip the balance in favor of the lower priced eggs or the cage free eggs. Brain activation in the dlPFC could serve as index of decision-making indifference. Activation in dlPFC has been previously associated with processing of uncertainty [14–16] and with making difficult decisions involving tradeoffs [6]. Thus, greater activation in dlPFC, to the extent that it reflects a measure of greater uncertainty and difficulty making a complex choice, is hypothesized to predict responsiveness to information.

To empirically explore the influence of dlPFC on valuation of a multi-attribute choice with conflicting individual attributes and the role of dlPFC in information response, we utilize an application for which there is much controversy and for which information has been shown to influence choice: animal welfare and food choice. Concerns about the impact of confined agricultural production systems on farm animal welfare have increased in recent decades. This is evident by California’s 2008 passing of the state-wide ballot initiative Proposition 2, the Prevention of Farm Animal Cruelty Act, establishing minimum space requirements for laying hens. Despite the popularity of legislation regulating confined production systems, however, consumers show less willingness or ability to pay for such practices in the marketplace, with fewer than 5% of eggs coming from cage-free systems [17]. Dissonance in buying preferences and voting behavior has important implications for egg producers, as it forces the adoption of production methods that consumers are not willing to support in the marketplace. The dissonance may arise from people having little knowledge about egg production methods and effective information campaigns from animal rights advocacy groups. For example, consumers believe a much higher share of eggs are produced using cage-free systems than actually are [17] and campaign advertising surrounding Proposition 2 led to an increase in demand for organic eggs [18].

Previous economic research has determined consumers’ WTP for eggs from various production methods [19–22], and examined the effects of information on WTP [23], however, little is known about *why* some people are more responsive to information than others. There is a need to better understand the factors affecting how people respond to campaign advertising and employing a neuroeconomic approach may be useful for gaining a better understanding.

In the present study, participants were placed in a magnetic resonance imaging (MRI) scanner and functional magnetic resonance imaging (fMRI) data were collected while participants made non-hypothetical choices between two options that varied by multi-attributes (i.e., production method and price) and single-attributes (i.e., production method or price). In the single-attribute choices, we expected participants to consistently choose the option that increased animal welfare or had a lower price. However, the multi-attribute choices (i.e., lower price combined with decreased animal welfare) posed a decisional conflict and produced uncertainty. Furthermore, we hypothesized that uncertainty—measured by response time—would be greater when making multi-attribute choices compared to single-attribute choices.

Although it is difficult to determine if dlPFC activation when making multi-attribute choices comes from valuation of options, comparison of options, or uncertainty in conflicting attributes, or possibly all of these, we hypothesized that activation in dlPFC would be greater when making multi-attribute choices with conflicting individual attributes. Activation in dlPFC likely varies by hemisphere and each hemisphere may provide unique information. Recent studies have suggested that a laterality effect may be present during certain gambling tasks and decision-making [24, 25]. Knoch et al. [26] found that decision-making was more risky after disruption of the right dlPFC (rdlPFC), but disruption of left dlPFC (ldlPFC) did not increase risky decision-making. Therefore, analysis were completed using activations from both ldlPFC and rdlPFC to account for any laterality effect that may be present.

After making non-hypothetical choices in response to these single- and multi-attribute options, participants were either shown a campaign advertisement in support or opposition of Proposition 2, and then repeated the non-hypothetical choices. We hypothesized that the proportion of times participants chose the multi-attribute option that involved increased animal welfare but a higher price would increase (decrease) after viewing the advertisement that supported (opposed) Proposition 2. Moreover, we hypothesized that increased activation in dlPFC while making multi-attribute choices prior to viewing a Proposition 2 campaign advertisement was indicative of information responsiveness.

## Materials and Methods

### Participants

The present study was approved by the Social Sciences Institutional Review Board of the University of Missouri-Kansas City (UMKC), as well as the Human Subjects Committee of the University of Kansas Medical Center (KUMC). All participants provided their written, informed consent to participate, the procedure for which was approved by the aforementioned organizations. A sample of 44 healthy, right-handed, English-speaking, adult participants (23 females; mean age = 29.6 ± 0.21, SEM; age range, 21–55 years) were recruited from the Kansas City metropolitan area to participate in an fMRI study. Exclusion criteria included current use of psychotropic medication, current or past substance abuse, diagnosis of severe psychopathology (e.g., depression, schizophrenia), and vegan diet. Motivational state varied and we assessed hunger immediately prior to the fMRI scan. Participants were paid $45 for their participation in the experiment and were also told that one of their choices was binding, and therefore left the experiment with one dozen eggs. While 50 participants completed the experiment, six participants were excluded from the analyses due to failure to follow or understand instructions for performing the food choice task. Thus, analyses were conducted using observations from 44 participants.

### Stimuli

Participants underwent two phases of fMRI scans while performing a food choice task—one functional scan before viewing a 30-second campaign advertisement and one functional scan after viewing a campaign advertisement. Participants were presented with the following instructions: “In this phase of the experiment, you will make a series of choices between two food products. To choose the option on the left, use your index finger. To choose the option on the right, use your middle finger. Please choose carefully, as you will receive one of the food products you choose at the end of the experiment. In the middle of this phase, there will be a brief pause while the scanner restarts. When you are ready, we will begin.”

**Fig 1:**
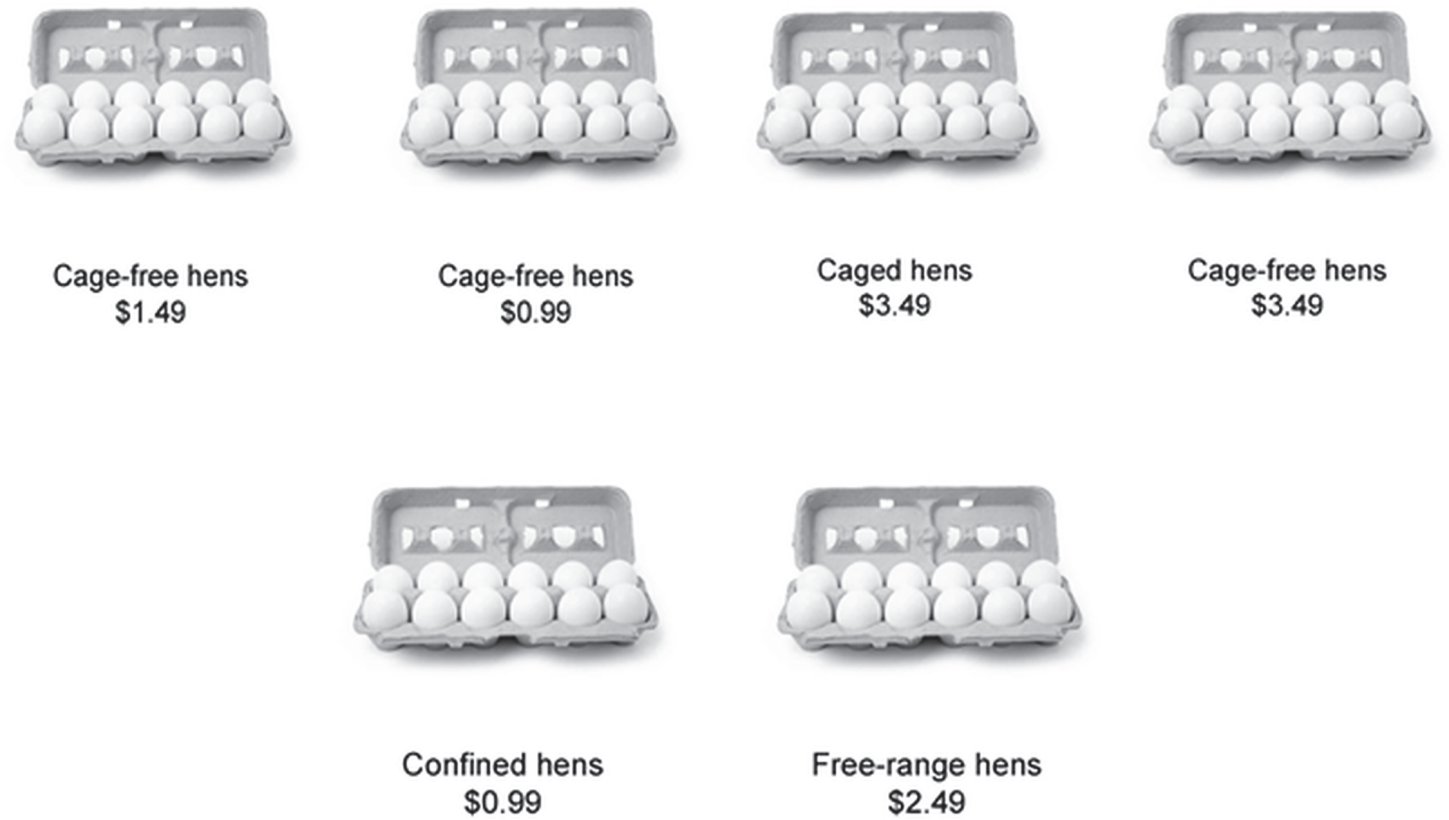
Examples of the three experimental conditions in the food decision-making task. a. Example of a price decision. b. Example of a production method decisions. c. Example of a combination decision.

**Fig 2:**
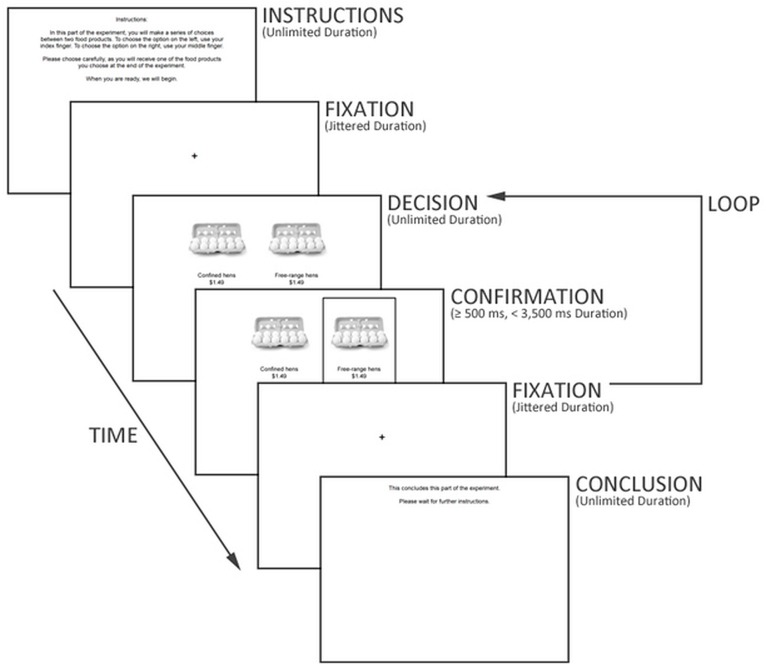
Timeline of food choice task.

The two options presented included an identical image of a dozen eggs accompanied by text indicating the production system and price for each option. Each option differed according to three experimental conditions: 1) a “method” condition, in which the method used to produce one option was “closed” (i.e., labeled “caged” or “confined”), and the method used to produce the other option was “open” (i.e., labeled “cage-free” or “free-range”), but the prices for both options were equal; 2) a “price” condition, in which the price of one option was higher than the other option but the production methods were identical; and 3) a “combination” condition, in which the production methods and prices of the two options differed in a manner that the open method was always accompanied with a higher price. Thus in the “combination” condition, participants were forced to make a tradeoff between animal welfare and price. Price began at “$0.99” and varied by $0.50 increments up to “$4.49.” Fig 1 illustrates examples of the three experimental conditions, while Fig 2 illustrates the timeline of the food choice task.

### Task

Participants made 84 choices during the first phase prior to viewing a video: 28 choices per experimental condition (i.e., combination, method, and price). The presentation order of the choices was randomized across respondents. The choices were made non-hypothetical by informing respondents that one of their choices would be randomly selected as binding and would actually be given to them at the conclusions of the experiment. Participants had an open-ended time period to make each choice, thus resulting in slightly different length of functional runs. If the participant chose in under 3,000 milliseconds, the participant’s choice was confirmed until 3,000 milliseconds had elapsed since the time the choice was presented, and then for an additional 500 milliseconds, if the choice took longer than 3,000 milliseconds, the choice was confirmed for an additional 500 milliseconds from the time of the choice. Average duration of response time was 790.76 milliseconds. Fixation intervals between decisions were jittered from zero to 13 seconds with an average duration of 2,865 milliseconds. The optimal stimulus and delay timing was determined using Analysis of Functional Neuroimages.

After undergoing the first phase, participants viewed a thirty-second video. Participants were randomly shown one of three videos; 16 participants viewed a campaign advertisement in support of Proposition 2 [27], 16 participants viewed a campaign advertisement in opposition to Proposition 2 [28], and 12 participants viewed control video that depicted a flowing stream. The campaign advertisements were actual commercials that aired in California prior to the vote on Proposition 2. Immediately following a video, the functional scan described previously was repeated so that there were two functional scans of 84 choices; 168 choices in total (84 prior to a video and 84 after a video).

### fMRI Data Acquisition

All fMRI scans were performed at the University of Kansas Medical Center’s Hoglund Brain Imaging Center on a 3-Tesla Siemens Skyra (Siemens, Erlangen, Germany) scanner. Participants’ heads were immobilized with head cushions. Following automated scout image acquisition and shimming procedures performed to optimize field homogeneity, a structural scan was completed. T1-weighted, three-dimensional, magnetization-prepared rapid acquisition with gradient echo (MPRAGE) structural images were acquired (repetition time/echo time [TR/TE] = 2300/2 ms, flip angle = 9°, field of view [FOV] = 256 x 256 mm, matrix = 256 x 192 mm, in-plane resolution = 1 x 1 mm, gap thickness = 0 mm, slice thickness = 1 mm). Then, two gradient-echo, blood oxygen level-dependent (BOLD) functional scans were acquired in fifty contiguous, oblique, 40° axial slices (TR/TE = 3000/25 ms, flip angle = 90°, FOV = 232 mm, matrix = 80 x 80 mm, in-plane resolution = 2.9 x 2.9 mm, gap thickness = 0 mm, slice thickness = 3 mm, 176 data points). To optimize the signal in prefrontal regions in the present study, and to minimize susceptibility artifacts, all participants were positioned such that the angle of the anterior commissure-posterior commissure (AC-PC) plane fell between 17° and 22° in scanner coordinate space, as verified by a localization scan. This careful positioning, utilized by Bruce and colleagues [29, 30], ensured the 40° acquisition angle was applied uniformly for all participants, again, minimizing susceptibility artifacts while standardizing the head positions of participants of divergent body sizes.

fMRI data were analyzed using BrainVoyager QX, version 2.4 (Brain Innovation, Maastricht, Netherlands, 2012). Preprocessing steps included trilinear, three-dimensional motion correction, sinc-interpolated slice scan time correction, two-dimensional spatial smoothing with a four-millimeter Gaussian filter, and high-pass filter temporal smoothing. Functional images were realigned to fit structural images obtained during each scanning session, then normalized to the BrainVoyager template image, which conforms to the space defined by Talairach and Tournoux’s [31] stereotaxic atlas. fMRI runs with greater than 3mm of motion were excluded from the analysis, this resulted in the exclusion of one fMRI run from one participant. Neural activation maps were analyzed using statistical parametric methods [32] included with the BrainVoyager QX software. Statistical contrasts of neural activation in the experimental conditions of interest (i.e., method, price, and combination conditions) were conducted using multiple-regression analysis. Regressors representing neural activation in these conditions, as well as regressors of non-interest (e.g., head motion), were modeled with a hemodynamic response filter. Group analysis was performed by entering data into the multiple-regression analysis using a random effects model. We chose to focus on a region of interest analysis using coordinates from a previous study of consumers viewing food products [13]. The coordinates chosen also mostly overlapped other studies examining dlPFC activation [6, 7]. This ROI analysis was performed using a cube centered in left dlPFC (x,y,z = -43, 13, 24) with a diameter of 10mm and a cube centered in right dlPFC (x,y,z = 41,25,33), also with a diameter of 10mm. Average percent signal change from across the cubes was extracted and imported into SPSS for statistical analyses for further behavioral analysis.

## Data Analysis and Results

### Behavioral Data Analysis and Results

In the single-attribute experimental conditions, the open option (cage-free; free range) was chosen for 99.9% of the choices in the method condition and the low price option was chosen for 98.6% of the choices in the price condition. This result confirms that people prefer open production to closed production methods and lower prices to higher prices; it also shows people were paying attention to the choices and taking the experimental task seriously.

**Fig 3:**
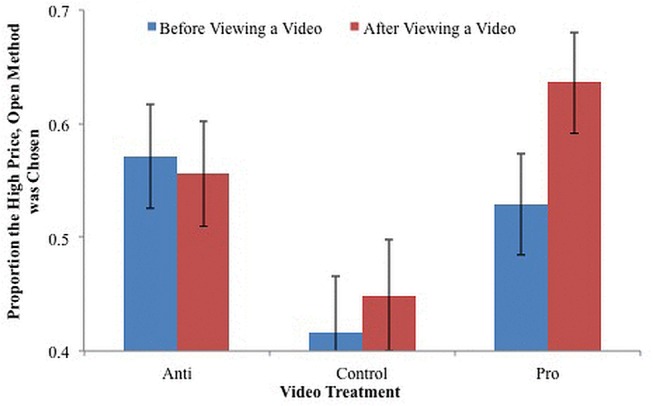
The effect of video information on the proportion the open method, high price option was chosen.

We focused analysis on combination decisions to investigate the effect of campaign advertising. Specifically, we were interested in the change in how often participants choose the open method, high price option instead of the closed method, lower price option after viewing a campaign advertisement. The proportion of choices in which the open method, high price option was chosen before and after viewing a video is shown in Fig 3. In the anti-Proposition 2 campaign advertisement treatment, participants chose the open method, high price option for 57% of the choices before viewing the campaign advertisement and for 56% of the choices after. The one-percent decrease was not a significant change (*t* = -0.73, *p* = 0.48), thus the anti-Proposition 2 campaign advertisement was not effective in changing behavioral choices. Participants in the control video treatment chose the open method, high price option for 42% and 44% of the choices before and after viewing the campaign advertisement, respectively. We did not expect the control video to affect choices and indeed the two-percent increase was not a significant change (*t* = 1.13, *p* = 0.28). The pro-Proposition 2 campaign advertisement, however, significantly increased the proportion of decisions for which the open method, high price option was chosen from 50% to 61% (*t* = 2.66, *p* = 0.02). That is, participants who viewed the pro-Proposition 2 campaign advertisement were more likely to choose the high price, open method option after viewing the campaign advertisement (i.e., they were more likely to be willing to pay a premium for cage free and free range eggs after viewing the video).

Uncertainty in decision-making between options that varied by multi-attributes (e.g., production method and price) compared to options that varied by a single-attribute (e.g., production method or price alone) has been measured by response time (RT) [6]. Combination choices were made between options with conflicting individual attributes, and those choices were likely more difficult relative to choices in the method and price conditions. We used an analysis of variance (ANOVA) comparison of the experimental condition choice RT means and performed orthogonal contrasts to examine pairwise differences between specific experimental condition choice RT means.

Before viewing a video, RT was significantly longer when making combination choices than method choices (*F* = 30.94, *p*<0.01) or price choices (*F* = 23.83, *p*<0.01). Using paired t-test, we found that RT for choices in all experimental conditions decreased after information for all video treatments (*p*<0.01 for all experimental conditions and video treatments). However, it is impossible to know how much of the decreased RT is attributable to video information as the choices made after information were repetitive. Differences in RT between multi-attribute and single-attribute choices decreased slightly after receiving video information, nevertheless, RT remained significantly longer when making combination choices compared to method choices (*F* = 4.96, *p* = 0.03) and price choices (*F* = 5.21, *p* = 0.02). These findings suggest the combination choices were more challenging and align with the findings of Kahnt et al. [6]. Further corroborating this hypothesis, RT was not significantly different when making choices between options that varied by only method or price before video information (*F* = 0.46, *p* = 0.50) or after video information (*F* = 0.00, *p* = 0.96).

### Imaging Data Analysis and Results

**Fig 4:**
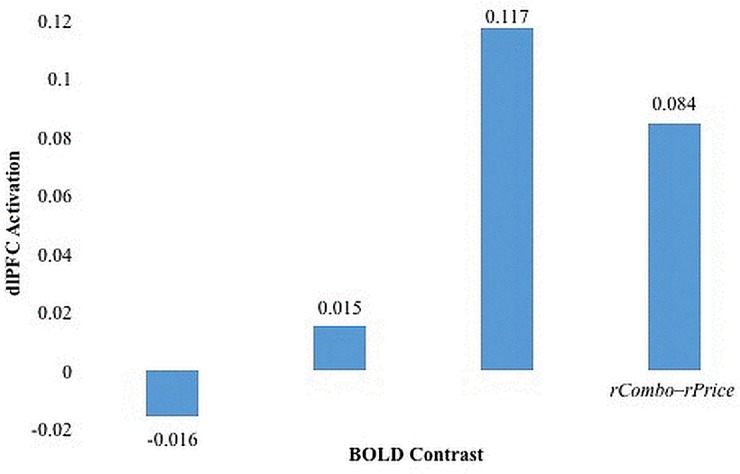
Means of the BOLD contrast variables.

All dlPFC activation examined was collected before participants viewed a video. To examine our hypotheses that dlPFC activation was greater when making multi-attribute choices with conflicting individual attributes, we contrasted percent blood-oxygen-level-dependent (BOLD) activation during combination decisions with BOLD activations during both the method and price choices. For example, BOLD activation in ldlPFC when making choices that varied by a single-attribute were subtracted from BOLD activation in ldlPFC when making choices that varied by both attributes to create the contrast variables *lCombo*–*lMethod* and *lCombo*–*lPrice*. That process was repeated for rdlPFC to create the contrast variables *rCombo*–*rMethod* and *rCombo*–*rPrice*. These activations were observed only before viewing a video. The mean values for the BOLD contrst variables are displayed in Fig 4.

Within-subject *t*-tests were used to test the null hypotheses that differences in activations for experimental conditions were equal to zero. Contrary to our hypothesis, participants did not exhibit greater activation in ldlPFC when making combination choices compared to either method choices (*t* = -0.64 *p* = 0.52) or price choices (*t* = 0.55, *p* = 0.58). However, confirming our hypothesis, activation in rdlPFC was significantly greater while making combination choices compared to both method choices (*t* = 2.88, *p*<0.01) and price choices (*t* = 2.50, *p* = 0.02). Therefore, in rdlPFC, there does appear to be more activation when making decisions between multi-attribute choices than single-attribute choices.

Change in proportion of times the open method, high price option was chosen before and after viewing a video signals a response to information. Correlation coefficients were estimated to examine the relationship between activation contrast variables and the change in proportion the option with the open method and high price was chosen for each video treatment. Coefficient estimates are shown in Table 1. Several activation contrast variables were significantly correlated in all video treatments. However, there does not appear to be a strong relationship between the proportion of open method, high price selection after viewing a video and activation contrast variables, as none of the coefficient estimates were significant. This result does not support our hypothesis that activation in dlPFC pre-video would indicate change in choice after viewing a video, at least linearly.

**Table 1:** Correlation Coefficients between Change in High Price, Open Method Proportion and dlPFC Activation Contrast Variables for each Video Treatment.

anti-Proposition 2 Video Treatment					
	*Change in High Price*, *Open Method Option*	*lCombo*–*lMethod*	*lCombo*–*lPrice*	*rCombo*–*rMethod*	*rCombo*–*rPrice*
*Change in High Price*, *Open Method Option*	1				
*lCCombo*–*lMethod*	-0.01	1			
	(0.97)				
*Combo*–*lPrice*	-0.40	0.55	1		
	(0.13)	(0.03)			
*rCombo*–*rMethod*	0.11	0.27	0.13	1	
	(0.68)	(0.31)	(0.62)		
*rCombo*–*rPrice*	0.04	0.24	0.40	0.68	1
	(0.89)	(0.37)	(0.12)	(0.00)	
Control Video Treatment					
	*Change in High Price*, *Open Method Option*	*lCombo*–*lMethod*	*lCombo*–*lPrice*	*rCombo*–*rMethod*	*rCombo*–*rPrice*
*Change in High Price*, *Open Method Option*	1				
*lCombo*–*lMethod*	0.13	1			
	(0.68)				
*lCombo*–*lPrice*	-0.26	0.47	1		
	(0.41)	(0.12)			
*rCombo*–*rMethod*	-0.35	0.73	0.35	1	
	(0.26)	(0.01)	(0.26)		
*rCombo*–*rPrice*	-0.12	0.59	0.39	0.71	1
	(0.71)	(0.04)	(0.22)	(0.01)	
pro-Proposition 2 Video Treatment					
	*Change in High Price*, *Open Method Option*	*lCombo*–*lMethod*	*lCombo*–*lPrice*	*rCombo*–*rMethod*	*rCombo*–*rPrice*
*Change in High Price*, *Open Method Option*	1				
*lCombo*–*lMethod*	-0.37	1			
	(0.16)				
*lCombo*–*lPrice*	-0.29	0.56	1		
	(0.28)	(0.02)			
*rCombo*–*rMethod*	0.04	0.51	0.00	1	
	(0.87)	(0.04)	(1.00)		
*rCombo*–*rPrice*	-0.01	0.56	0.52	0.66	1
	(0.98)	(0.03)	(0.04)	(0.01)	

Note: Correlation coefficients were estimated using 16, 12, 16 observation for the anti-Proposition 2, control, and pro-Proposition 2 video treatments, respectively. *P*-values are in parenthesis.

To further explore these results at the disaggregate choice level, a binary logistic regression model was estimated to further analyze the effects of pre-video activation in dlPFC on choice before and after viewing a video in the combination condition. Subjects made 28 choices in the combination condition before viewing a video and another 28 afterwards. Therefore, there were 56 observations for each of the 44 participants. The dependent variable was equal to one if a subject chose the open method, high price option, and zero otherwise. Thus, the dependent variable indicates whether a subject was willing to pay a premium for an open production method option for a given choice.

Explanatory variables for the logistic regression model included: BOLD activation contrasts; indicator variables for the anti-Proposition 2 campaign advertisement (*Anti*) and pro-Proposition 2 campaign advertisement (*Pro*); an indicator variable for choices made after viewing a video (*After*); two-way interactions between *After* and BOLD activation contrasts; two-way interactions between *After* and campaign advertisement indicator variables; and three-way interaction between *After*, BOLD activation contrasts, and campaign advertisement indicator variables. BOLD activation contrasts were included to determine the effect of pre-video dlPFC activation on the proportion the open method, high price option was chosen. *Anti* and *Pro* were included to account for variation in the groups of subjects randomly assigned to video treatments. Two-way interactions between *After* and BOLD activation contrasts were included to determine the effect of pre-video dlPFC activation on the proportion the open method, high price option was chosen after viewing a video. Two-way interactions between *After* and campaign advertisement indicator variables were included to determine the effect of the Proposition 2 videos on the proportion the open method, high price option. Three-way interaction between *After*, BOLD activation contrasts, and video campaign advertisement indicator variables were included to determine the effect of pre-video dlPFC activation on the proportion the open method, high price option was chosen after Proposition 2 videos.

Standard errors were corrected for repeated measures across participants. Importantly, the BOLD activations are all measured prior to viewing a video. Thus, significant interactions between BOLD activations and *After* would support the hypothesis that pre-video dlPFC activation predicts responsiveness to information.

Estimation results from the logistic regression are shown in Table 2. None of the coefficient estimates for the activation variables were significant before viewing a video. This indicates that activation in ldlPFC and rdlPFC, when making combination choices relative to method and price choices, did not affect the probability that a subject chose the open method, high price option prior to receiving information. *Anti* and *Pro* were not significant; indicating that participants were randomly assigned to video information treatments with respect to the probability of choosing the open method, high price option

**Table 2:** Logistic Regression Estimation Results.

	Dependent Variable: *P*(*High Price*, *Open Method Option = 1*)		
Explanatory Variables	Coefficient Estimate	Standard Error	p-Value	
Intercept	-0.707	0.486	0.146	
*lCombo*—*lMethod*	-2.379	1.621	0.142	
*lCombo*—*lPrice*	1.824	1.469	0.214	
*rCombo*—*rMethod*	0.669	1.505	0.657	
*rCombo*—*rPrice*	1.046	1.791	0.559	
*Anti*	0.875	0.566	0.122	
*Pro*	0.474	0.596	0.427	
*After*	-1.114*	0.609	0.067	
*After*(*lCombo*—*lMethod*)	-3.646	4.375	0.405	
*After*(*lCombo*—*lPrice*)	-2.819	2.812	0.316	
*After*(*rCombo*—*rMethod*)	5.462***	1.937	0.005	
*After*(*rCombo*—*rPrice*)	-0.607	2.550	0.812	
*AfterAnti*	1.070*	0.650	0.100	
*AfterPro*	1.400**	0.654	0.032	
*AfterAnti*(*lCombo*—*lMethod*)	5.480	5.333	0.304	
*AfterAnti*(*lCombo*—*lPrice*)	3.982	3.881	0.305	
*AfterAnti*(*rCombo*—*rMethod*)	-10.472***	2.104	<.001	
*AfterAnti*(*rCombo*—*rPrice*)	6.928**	3.448	0.045	
*AfterPro*(*lCombo*—*lMethod*)	-0.723	5.555	0.897	
*AfterPro*(*lCombo*—*lPrice*)	1.582	3.753	0.674	
*AfterPro*(*rCombo*—*rMethod*)	-6.699**	3.369	0.047	
*AfterPro*(*rCombo*—*rPrice*)	1.159	3.849	0.763	
Log Likelihood	-1705			

Note: Estimates are from a binary logistic regression using based on 28 choices from 44 participants. Standard errors are clustered at the subject-level. Single, double, and triple asterisks (*, **, ***) indicate statistical significance at the 10%, 5%, and 1% level.

However, *After* was significant which indicated campaign advertising changed the probability of choosing the open method, high price option when accounting for variation in dlPFC activation. The interactions of *Anti* and *Pro* with *After* were also significant. The coefficient estimates for *AfterAnti* and *AfterPro* were both positive because the estimates are relative to the control group. The control group was less likely to choose the open method, high price option in general, as illustrated by Fig 3.

Activation in rdlPFC before campaign advertising signals change in the probability of choosing the open method, high price option after viewing campaign advertising. This was most evident when comparing BOLD activations during the combination and method conditions, as coefficient estimates for interactions between *rCombo*–*rMethod* and *After* were significant for both the *Anti* and *Pro* video treatments. Both coefficient estimates were negative, indicating that participants with higher *rCombo*–*rMethod* BOLD contrasts were less likely to choose the open method, high price option after receiving information. The BOLD contrast variable *rCombo*–*rPrice* was significant when interacted with *After* for the anti-Proposition 2 video treatment. The coefficient estimate was positive, indicating that participants with higher *rCombo*–*rPrice* BOLD contrasts were more likely to choose the open method, high price option after receiving information. These results confirm the hypothesis that increased activation in dlPFC while making multi-attribute choices prior to viewing a Proposition 2 campaign advertisement was indicative of information responsiveness.

**Fig 5:**
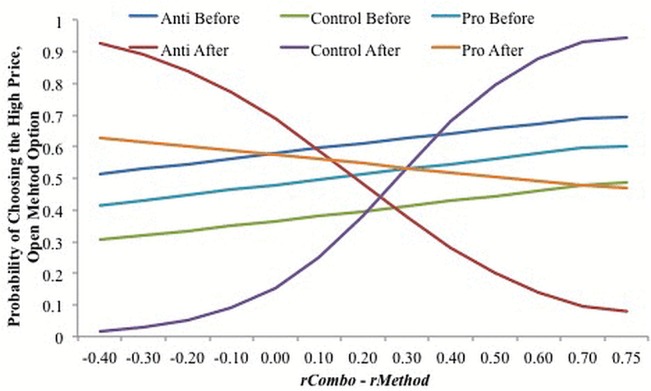
The effect of BOLD contrast variable rCombo–rMethod on the probability of choosing open method, high price option before and after video information.

Fig 5 shows how varying levels of the BOLD contrast *rCombo*–*rMethod* affects the probability of choosing the open method, high price while holding all other variables constant. Prior to viewing a video, participants in the anti-Proposition 2 and Control video treatments were most and least likely to choose open method, high price option, respectively. For the lowest values of *rCombo*–*rMethod*, the anti and pro-Proposition 2 campaign advertisements increased the probability of choosing the high price, open method option; however, the probability of choosing the open method, high price option decreased as *rCombo*–*rMethod* increased. The effect was opposite in the Control video treatment. It is possible that the Control video, that depicted a flowing stream, had an unanticipated effect on decisions, perhaps cueing a desire for “naturalness”.

## Discussion

This study used fMRI to examine the role of bilateral dlPFC activation in valuation of a non-hypothetical decision between multi-attribute choices. Results suggest rdlPFC had greater activation when making multi-attribute choices compared to single-attribute choices. This result is consistent with previous research that indicated dlPFC had a role in valuation [5, 9, 31] and integrated multiple value predictions [6].

Using response time values, we demonstrated it was more difficult for participants to make multi-attribute choices compared to single-attribute choices. Increased RT and rdlPFC activation while making multi-attribute choices seem to imply that participants were contemplating the tradeoff between animal welfare and price. If a participant was not concerned with one attribute, whether it be animal welfare or price, the options would be reduced to a single-attribute choice.

Given participant concern about both attributes, the longer RT and greater activation in the dlPFC may suggest that there was more uncertainty in the valuation for multi-attribute choices with conflicting individual attributes. If so, that would confirm Kahnt et al. [6]. Nevertheless, it confirmed the connection between dlPFC activation and uncertainty concluded in past studies [14–16].

This study also sought to determine if dlPFC activation while making decisions prior to viewing a video indicated how an individual may response to a video. Advertisements from California’s Proposition 2 in 2008, as well as a control video, were used because the choice options were eggs that varied by production method, as well as price. The results here suggest that the pro-Proposition 2 video was effective in persuading consumers—confirming Lusk’s [18] work that determined Proposition 2 advertising increased consumer demand for organic eggs. The anti-Proposition 2 video and, as expected, the control video was not effective in changing consumer behavior. Proposition 2 passed with 63% of voters voting in favor of increasing animal confinement space. Thus, it is possible voters’ were similarly persuaded in the voting booth as in the marketplace. Although participants were randomly assigned to video treatments, the average participant in the control treatment chose the open method, high price option significantly fewer times before and after information.

There was a relationship between activation in rdlPFC before viewing a video and choices after. However, there was no relationship between ldlPFC activation before viewing a video and subsequent choices. This result suggests a laterality effect may be present. Specifically, greater differences between combination and method activation in rdlPFC increased the likelihood of consumers choosing the open method, higher priced option was chosen after video information. This effect was obvious for the anti-Proposition 2 and control videos. Thus, large differences between rdlPFC activation when making multi-attribute choices (i.e., choices were a tradeoff between animal welfare and price was required) and single-attribute choices (i.e., choices that varied by animal welfare alone) may signal uncertainty and indicate a greater response to subsequent information.

People with more uncertainty—defined by larger differences in activation—were more persuaded by information, even when there was not an obvious connection between the information and the choice (i.e., the control video). While the effect of uncertainty was opposite for the control video—the proportion the open method, high price option was chosen increased with *rdlPFC*_*Combo*_–*rdlPFC*_*Method*_—the average participant in the control treatment was less likely to choose the open method, high price options before information; therefore, participants in the control treatment experiencing uncertainty may have also responded differently to information.

This study was, to a degree, limited by sample size, notably when estimating correlation coefficients, as the sample was split into three video treatments to include a control group. Future research could supplement the current study by examining the effects of different information and determining if other neural areas indicate responsiveness to information.
